# Supplementary material for: Violation of the constant genetic effect assumption can result in biased estimates for non-linear Mendelian randomization
Source: Hum Hered. Author manuscript; Available in PMC 2023 Nov 1. (PMC10614256; doi:10.1159/000531659)
Supplement: Supplementary Material [file EMS188505-supplement-Supplementary_Material.pdf]

Supplementary Material

We conducted a more systematic simulation study to investigate the degree of potential bias in the residual versus doubly-ranked method. We simulated data according to the same model as in Scenario 1 of Simulation study 2 from the main text, except varying the strength of the instrument and the variability of its effects on the exposure:

$$\begin{aligned}
g_i &\sim \mathcal{N}(0,1)\\
u_i &\sim \mathcal{N}(0,1)\\
\epsilon_i &\sim \mathcal{N}(0,1)\\
\alpha_i &\sim \mathcal{N}(\gamma_0 + \gamma_1 \times \epsilon_i, 0.1^2) \text{ independently}\\
x_i &= \alpha_i \times g_i + u + \epsilon_i
\end{aligned}$$

where γ_0 represents the average strength of the instrument, and γ_1 represents its variability. In scenarios 1, 2, and 3, we set $\gamma_0 = +0.2$; in scenarios 4, 5, and 6, we set $\gamma_0 = +0.3$; and in scenarios 7, 8, and 9, we set $\gamma_0 = +0.2$. In scenarios 1, 4, and 7, we set $\gamma_1 = +0.05$; in scenarios 2, 5, and 8, we set $\gamma_1 = +0.1$; and in scenarios 3, 6, and 9, we set $\gamma_1 = +0.15$. We generated 1000 datasets of 10 000 individuals for each scenario. We estimated the correlation between the genetic instrument G and the confounder U in each of 10 strata defined using the residual and doubly-ranked methods.

Results are shown in Supplementary Table A1. We see that the mean correlations in scenarios 1, 4, and 7 were similar for both methods. Similarly the mean correlations were similar in scenarios 2, 5, and 8; and scenarios 3, 6, and 9. In contrast, mean correlations were progressively higher across each set of three scenarios (1, 2, and 3; 4, 5, and 6; 7, 8, and 9) as the instrument variability was increased. This indicates that bias in Mendelian randomization estimates is dependent on the variability of the genetic effects on the exposure, not their strength.

In each scenario, the mean correlation was two times stronger for the residual method compared with the doubly-robust method, indicating that bias due to variability in the genetic effect on the exposure will be greater on average for the residual method. However, while this result held on average, in some simulated datasets correlations were stronger in the doubly-ranked method.

Residual method									
Stratum	Scenario 1	2	3	4	5	6	7	8	9
1	0.065	0.126	0.184	0.064	0.126	0.184	0.064	0.126	0.183
2	0.050	0.098	0.146	0.050	0.098	0.147	0.050	0.098	0.146
3	0.032	0.064	0.097	0.032	0.065	0.097	0.033	0.065	0.097
4	0.019	0.037	0.056	0.019	0.037	0.056	0.019	0.038	0.056
5	0.006	0.012	0.018	0.006	0.012	0.019	0.006	0.012	0.019
6	-0.006	-0.013	-0.018	-0.006	-0.012	-0.018	-0.006	-0.012	-0.019
7	-0.019	-0.037	-0.056	-0.019	-0.037	-0.056	-0.019	-0.037	-0.056
8	-0.032	-0.065	-0.097	-0.033	-0.064	-0.097	-0.032	-0.064	-0.098
9	-0.050	-0.099	-0.146	-0.049	-0.098	-0.146	-0.050	-0.098	-0.146
10	-0.064	-0.125	-0.183	-0.065	-0.126	-0.183	-0.065	-0.125	-0.184
Doubly-ranked method									
Stratum	Scenario 1	2	3	4	5	6	7	8	9
1	0.033	0.064	0.096	0.032	0.064	0.096	0.032	0.065	0.096
2	0.022	0.044	0.066	0.022	0.044	0.066	0.022	0.045	0.066
3	0.015	0.029	0.043	0.015	0.030	0.044	0.015	0.030	0.044
4	0.009	0.017	0.025	0.008	0.017	0.026	0.009	0.017	0.026
5	0.003	0.005	0.008	0.002	0.006	0.009	0.003	0.006	0.008
6	-0.003	-0.006	-0.008	-0.003	-0.006	-0.008	-0.003	-0.006	-0.009
7	-0.009	-0.017	-0.025	-0.009	-0.017	-0.025	-0.009	-0.017	-0.025
8	-0.015	-0.030	-0.044	-0.015	-0.029	-0.044	-0.015	-0.029	-0.044
9	-0.022	-0.044	-0.066	-0.022	-0.044	-0.066	-0.022	-0.044	-0.066
10	-0.032	-0.064	-0.096	-0.033	-0.065	-0.096	-0.033	-0.064	-0.097

Supplementary Table A1: Mean correlations between the genetic instrument and confounder within strata in the additional simulation study for the residual and doubly-ranked methods.

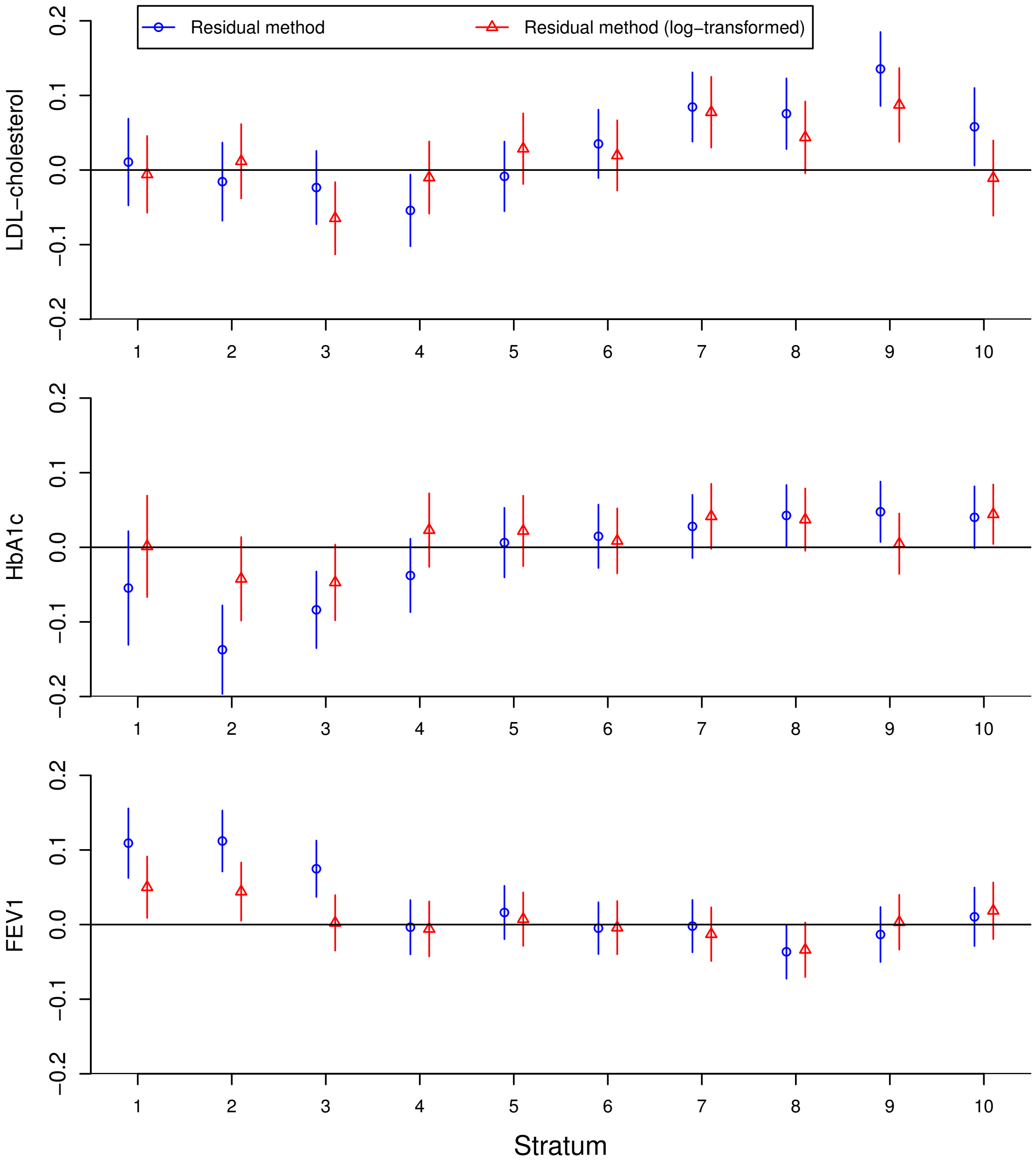

Supplementary Figure A1: Genetic associations with covariates in strata defined by the residual method for untransformed 25(OH)D (blue circles) and log-transformed 25(OH)D (red triangles). Estimates represent associations with the covariate in standard deviation units per unit change in the genetic instrument, corresponding to a standard deviation change in 25(OH)D concentrations. Error bars are 95% confidence intervals.
